# Long‐term dementia risk prediction by the LIBRA score: A 30‐year follow‐up of the CAIDE study

**DOI:** 10.1002/gps.5235

**Published:** 2019-12-06

**Authors:** Kay Deckers, Mariagnese Barbera, Sebastian Köhler, Tiia Ngandu, Martin van Boxtel, Minna Rusanen, Tiina Laatikainen, Frans Verhey, Hilkka Soininen, Miia Kivipelto, Alina Solomon

**Affiliations:** ^1^ Alzheimer Centrum Limburg, School for Mental Health and Neuroscience (MHeNS) Maastricht University Maastricht The Netherlands; ^2^ Institute of Clinical Medicine/Neurology University of Eastern Finland Kuopio Finland; ^3^ Public Health Promotion Unit National Institute for Health and Welfare Helsinki Finland; ^4^ Division of Clinical Geriatrics, Center for Alzheimer Research, Department of Neurobiology, Care Sciences, and Society Karolinska Institutet Stockholm Sweden; ^5^ Institute of Public Health and Clinical Nutrition University of Eastern Finland Kuopio Finland; ^6^ Hospital District of North Karelia Joensuu Finland; ^7^ Neurocenter, Department of Neurology Kuopio University Hospital Kuopio Finland; ^8^ Ageing Epidemiology (AGE) Research Unit, School of Public Health Imperial College London London United Kingdom

**Keywords:** cognitive aging, cohort study, dementia, epidemiology, lifestyle, prevention, risk factors

## Abstract

**Objective:**

As no causal treatment for dementia is available yet, the focus of dementia research is slowly shifting towards prevention strategies. Therefore, this study aimed to examine the predictive accuracy of the “LIfestyle for BRAin Health” (LIBRA) score, a weighted compound score of 12 modifiable risk and protective factors, for dementia and mild cognitive impairment (MCI) in midlife and late‐life, and in individuals with high or low genetic risk based on presence of the apolipoprotein (APOE) ε4 allele.

**Methods:**

The LIBRA score was calculated for participants from the Finnish Cardiovascular Risk Factors, Aging and Dementia (CAIDE) population‐based study examined in midlife (n = 1024) and twice in late‐life (n = 604) up to 30 years later. Diagnoses of MCI and dementia were made according to established criteria. Cox proportional hazards models were used to assess the association between LIBRA and risk of dementia and MCI in models adjusted for sex and education (age as timescale).

**Results:**

Higher midlife LIBRA scores were related to higher risk of dementia (hazard ratio [HR] = 1.27; 95% confidence interval [CI], 1.13‐1.43) and MCI (unadjusted model: HR = 1.12; 95% CI, 1.03‐1.22) up to 30 years later. Higher late‐life LIBRA scores were related to higher risk of MCI (HR = 1.11; 95% CI, 1.00‐1.25), but not dementia (HR = 1.02; 95% CI, 0.84‐1.24). Higher late‐life LIBRA scores were related to higher dementia risk among apolipoprotein E (APOE) ε4 non‐carriers.

**Conclusions:**

Findings emphasize the importance of modifiable risk and protective factors for dementia prevention.

**Key points**

1. Midlife and late‐life are suitable life periods to target health and lifestyle factors to reduce dementia risk.
2. The “LIfestyle for BRAin health” (LIBRA) score may be useful for educational/motivational purposes by emphasizing areas amenable topreventive lifestyle measures, and for identifying at‐risk individuals whomay benefit from lifestyle interventions.

## 1 INTRODUCTION

Dementia is one of the core challenges facing our aging society.^1^ Prevention is crucial given that there are no treatments available to stop or reverse dementia.^2^ For this, evidence‐based preventive strategies are needed, focusing on modifiable risk factors.^3^, ^4^, ^5^ Such strategies should include the early identification of persons at risk for dementia, eg, in midlife, in order to target risk factors before irreparable brain damage and cognitive symptoms occur. The “LIfestyle for BRAin Health” (LIBRA) score was developed based on a systematic literature review and expert consensus study.^6^ It consists of modifiable risk and protective factors that are promising targets for preventive strategies and thus reflects an individual's prevention potential for dementia. So far, LIBRA has been shown to explain variance in cognitive functioning and dementia risk in various population‐ and patient‐based prospective cohort studies.^7^, ^8^, ^9^, ^10^, ^11^ Yet, more research is needed into the predictive validity of LIBRA: (a) for other outcome measurements such as mild cognitive impairment (MCI), (b) in an aging cohort (followed from midlife to late‐life), (c) in long‐term cohort studies where follow‐up may be decades later, and (d) in interaction with genetic risk (eg, apolipoprotein E [APOE] ε4 genotype).

Therefore, the overall aim of the present study was to investigate the predictive validity of the LIBRA score in the longitudinal population‐based Cardiovascular Risk Factors, Aging and Dementia (CAIDE) study.^12^ The first aim was to investigate the performance of the LIBRA score, measured in the same individuals, midlife (40‐50 years) and late‐life (65‐79 years) for predicting the risk of incident dementia and MCI up to 30 years later. The second aim was to investigate potential differences between persons with high and low genetic risk for dementia (APOE ε4 carriers versus non‐carriers) regarding the relations of the LIBRA score with dementia and MCI risk.

## 2 MATERIALS AND METHODS

### 2.1 The CAIDE study

CAIDE participants were randomly selected from four independent population‐based samples of the North Karelia Project and the FINMONICA study. Participants were examined in midlife^13^, ^14^, ^15^ in 1972, 1977, 1982, or 1987. In 1998, a random sample of 2000 individuals aged 65 to 79 years from the towns and surroundings of Kuopio and Joensuu in Eastern Finland was invited for the first re‐examination.^12^ Of these, 1449 persons (72.5%) participated. A second re‐examination took place between 2005 and 2008. Of the initial 2000 individuals, 1426 were still alive and living in the target areas in 2005, and 909 (63.7%) attended the re‐examination (Figure S1). In total, 1511 participants attended at least one re‐examination, and 750 attended both re‐examinations, with completed cognitive assessments. Mean follow‐up time (SD) from midlife was 20.9 (4.9) years until the first re‐examination, and 28.9 (5.0) years until the second re‐examination. The study was approved by the local ethics committee of Kuopio University and Kuopio University Hospital, and written informed consent was obtained from all participants.

### 2.2 Assessment of MCI and dementia

In both re‐examinations, cognitive status was assessed with a three‐step protocol (screening, clinical, and differential diagnostic phase). Individuals scoring less than or equal to 24 on the Mini‐Mental State Examination (MMSE)^16^ at screening were referred for additional clinical assessments. In 2005 to 2008, individuals with less than or equal to 24 points or a decline greater than or equal to three points on MMSE, less than 70% delayed recall in the CERAD word list,^17^ or an informant expressing concerns about the participant's cognition were referred for more assessments. Both re‐examinations had a clinical phase with detailed neuropsychological and medical assessments and a differential diagnostic phase. A review board consisting of a senior neurologist, senior neuropsychologist, study physician, and study neuropsychologist ascertained the final diagnosis based on all available information. In both re‐examinations, diagnosis of MCI and dementia were made according to established criteria.^18^, ^19^, ^20^

### 2.3 Design of the present study

**Figure 1:**
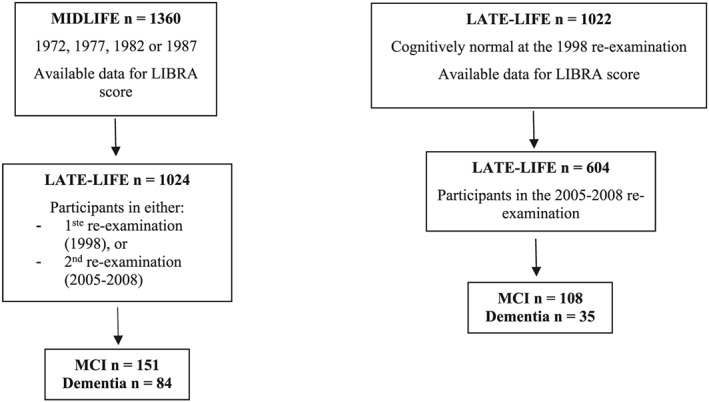
Study design

For midlife LIBRA score analyses, the study population included 1024 CAIDE participants with available data for the midlife LIBRA score and who attended at least one re‐examination with completed cognitive assessments (Figure 1). Outcomes were incident dementia (n = 84) or MCI (n = 151) as diagnosed at the CAIDE re‐examination visits.

For late‐life LIBRA score analyses, the study population included 604 individuals with available 1998 data for the late‐life LIBRA score, who were cognitively normal at the 1998 re‐examination (ie, no dementia or MCI), and who returned for the 2005 to 2008 re‐examination. Outcomes were incident dementia (n = 35) or MCI (n = 108) as diagnosed at the 2005 to 2008 re‐examination.

### 2.4 LIBRA score

The LIBRA score was developed after triangulation of results from a systematic literature review on risk and protective factors for dementia and an expert consensus study,^6^ as part of the European (FP7) INnovative, Midlife INtervention for Dementia Deterrence (In‐MINDD) project.^21^ It consists of 12 modifiable risk and protective factors that can be targeted by tailored lifestyle interventions and primary prevention. Risk factors are coronary heart disease, diabetes, hypercholesterolemia, hypertension, depression, obesity, smoking, physical inactivity, and renal disease. Protective factors are low‐to‐moderate alcohol use, high cognitive activity, and healthy diet. A weight is assigned to each factor, based on the factor's relative risk (Table S1).^6^ Weights are then standardized and summed up to yield the final LIBRA score (range from −5.9 to +12.7), with higher scores indicating greater risk. A modified version of the LIBRA score was developed for the purpose of validation in older cohorts. It consists of 10 factors, excluding the risk factors obesity and hypertension, since these are considered to be major risk factors in midlife only.^3^, ^22^, ^23^, ^24^

### 2.5 Assessment of LIBRA

At the midlife examination, assessments and survey methods were standardized and adhered to international guidelines and the World Health Organization (WHO) (Multinational MONItoring of trends and determinants in CArdiovascular [MONICA] disease) protocol.^25^ CAIDE re‐examination surveys were similar to midlife. Surveys involved self‐administered questionnaires on medical history, sociodemographic and psychological factors, and health‐related behaviors. A trained nurse verified the answers, and measured height, weight and blood pressure. A venous blood sample was taken to determine serum total cholesterol. APOE genotype was determined from blood leucocytes using polymerase chain reaction and HhaI digestion.^26^

In CAIDE, data were available for all LIBRA factors, except for cognitive activity. Factors were dichotomized based on previously used cut‐offs (Table S1). Hypertension was defined as a systolic blood pressure ≥ 140 mmHg or diastolic blood pressure ≥ 90 mmHg. Participants were classified as obese if their body mass index (BMI) exceeded 30 kg/m^2^. The cut‐off point for hypercholesterolemia was greater than or equal to 6.5 mmol/L. Diabetes and coronary heart disease were based on self‐reports of diagnoses made by a physician and diagnoses from the Finnish Hospital Discharge Register. Renal disease was based solely on diagnoses from the Finnish Hospital Discharge Register. A cut‐off point for depressive symptoms was created based on the sum scores of two questions related to feelings of hopelessness.^27^ Persons who engaged in physical activity at least twice a week, lasting at least 20 to 30 minutes each occasion, and causing sweating and breathlessness, were regarded as physically active. Low‐to‐moderate alcohol consumption was based on categorized frequency of alcohol use. For smoking, participants were divided into ever smokers and never smokers. Since information on diet was available for only a small group of approximately 240 participants in midlife, analyses including diet were conducted separately. Adherence to a healthy diet was based on previously used cut‐offs of the CAIDE Healthy Diet Index.^28^ Observed LIBRA scores ranged from −2.7 to +12.7.

### 2.6 Statistical analysis

To examine differences in risk factors and demographic variables between participants with incident dementia, MCI and controls, one‐way analysis of variance (ANOVA) and *χ* ^2^ tests were used. Cox proportional hazard regression models were used to test associations between the LIBRA score and dementia or MCI risk. Harrell concordance rate (C statistic) for censored data was calculated to examine predictive accuracy. The C‐statistic indicates the probability that a randomly selected participant who developed the outcome (dementia or MCI) had a higher risk score than a participant who did not develop the outcome. It is equal to the area under the Receiver Operating Characteristic curve and ranges from 0.5 to 1. Model 1 was unadjusted (except for age as the time scale, see below), and model 2 was adjusted for the covariates sex and education. In addition, we tested for a multiplicative interaction between LIBRA score and APOE genotype (ε4 carriers versus non‐carriers). All analyses were done in Stata 14 (StataCorp LP, TX), and the level of statistical significance was *P* < .05.

For midlife LIBRA analyses, *stcox* was used with age as time scale and age at first assessment (midlife) as origin. Right censoring was defined as the age at the first dementia diagnosis in CAIDE re‐examinations or end of study (date of last available CAIDE re‐examination). The same approach was used in analyses with MCI as outcome (ie, considering date of first MCI diagnosis). The proportional hazard assumption was assessed based on the Schoenfeld residuals.

For late‐life LIBRA analyses, *stcox* was used, with age as time scale and age during the later assessment wave in 1998 as origin. Censoring age at follow‐up was defined analog to the midlife analysis at the 2005 to 2008 CAIDE re‐examination.

## 3 RESULTS

### 3.1 Population characteristics

Population characteristics are shown in Table 1. As expected, individuals with incident dementia were older, had a lower education level, and were more often APOE ε4 carriers (all *P* < .01). Sex distribution was not significantly different between diagnostic groups.

**Table 1:** Characteristics of the study population

	Study Population				
	n (Control/MCI/Dementia)	Control	Incident MCI	Incident Dementia	*P* Value
Sex (women)	793/147/84	61.0%	60.5%	58.3%	.89
Education, y	789/146/84	8.9 (3.5)	7.8 (2.7)	8.0 (3.5)	<.001
APOE4 carriers	781/145/81	33.9%	37.2%	54.3%	<.01
**Midlife**					
Age	793/147/84	47.8 (4.7)	48.2 (4.8)	50.6 (4.8)	<.001
Total cholesterol, mmol/L	793/147/84	6.7 (1.2)	6.9 (1.1)	6.9 (1.1)	.39
Systolic blood pressure, mmHg	793/147/84	142.3 (18.8)	144.6 (22.1)	149.7 (22.3)	<.01
Diastolic blood pressure, mmHg	793/147/84	89.6 (10.6)	90.7 (10.7)	93.4 (10.3)	<.01
Body mass index	793/147/84	26.0 (3.5)	26.7 (3.6)	27.2 (3.9)	<.01
LIBRA score	793/147/84	3.1 (1.8)	3.3 (1.6)	4.0 (2.1)	<.001
LIBRA score including diet	184/35/20	2.6 (2.2)	2.9 (1.8)	3.6 (2.1)	.13
Low/moderate alcohol use	793/147/84	43.6%	35.4%	38.1%	.13
Smoking	793/147/84	47.0%	40.8%	42.86	.32
Physical inactivity	793/147/84	60.1%	58.5%	69.0%	.24
Depressive symptoms	793/147/84	8.7%	12.9%	13.1%	.15
Coronary heart disease	793/147/84	4.8%	6.1%	4.8%	.79
Diabetes mellitus	793/147/84	0.8%	0.7%	0.0%	.73
High cholesterol	793/147/84	52.8%	60.5%	65.5%	<.05
Obesity	793/147/84	11.7%	13.6%	25.0%	<.01
Hypertension	793/147/84	63.7%	63.3%	80.9%	<.01
Chronic kidney disease	793/147/84	0 %	0 %	0 %	‐
Healthy diet	184/35/20	43.5%	40.0%	25.0%	.27
**late‐life (1998)**					
Age	461/108/35	69.8 (3.4)	70.6 (3.2)	72.4 (4.3)	<.001
Total Cholesterol, mmol/L	461/108/35	5.8 (1.0)	5.8 (1.0)	5.5 (1.1)	.22
Systolic blood pressure, mmHg	461/108/35	150.5 (22.9)	151.1 (21.9)	140.2 (23.0)	<.05
Diastolic blood pressure, mmHg	461/108/35	81.3 (10.6)	81.2 (10.9)	76.1 (10.4)	<.05
Body mass index	461/108/35	27.6 (3.9)	28.3 (4.0)	26.7 (4.0)	.08
LIBRA score	461/108/35	2.7 (1.7)	3.1 (1.6)	2.8 (1.9)	.05
Low‐moderate alcohol use	461/108/35	38.0%	34.3%	48.6%	.31
Smoking	461/108/35	35.4%	32.4%	31.43%	.78
Physical inactivity	461/108/35	17.6%	24.1%	25.7%	.18
Depressive symptoms	461/108/35	7.6%	11.1%	17.1%	.10
Coronary heart disease	461/108/35	25.4%	36.1%	45.7%	<.01
Diabetes mellitus	461/108/35	8.5%	6.4%	11.4%	.62
High cholesterol	461/108/35	24.3%	20.4%	28.6%	.55
Obesity	461/108/35	22.6%	32.4%	22.9%	.10
Hypertension	461/108/35	69.4%	76.8%	48.6%	<.01
Chronic kidney disease	461/108/35	0 %	0 %	0 %	‐

*Note*. Values are means (SD) unless otherwise specified.

Abbreviations: APOE, apolipoprotein; LIBRA, LIfestyle for BRAin health; MCI, mild cognitive impairment.

Midlife LIBRA scores were higher in participants who subsequently developed dementia (total population: n = 1024; mean LIBRA score [SD]: 3.22 [1.82]; range: −1.0 to 9.3). From the list of individual LIBRA factors, hypertension (elevated systolic and diastolic blood pressure), obesity (high BMI), and high cholesterol were significantly more common among the incident dementia group.

Differences in late‐life LIBRA scores between diagnostic groups were less pronounced (total population: n = 604; mean LIBRA score (SD): 2.76 (1.70); range: −1.0 to 7.5). The incident dementia group included a higher percentage of individuals with coronary heart disease and a lower percentage of individuals with hypertension (lower systolic and diastolic blood pressure).

### 3.2 Midlife LIBRA and incident dementia and MCI

**Figure 2:**
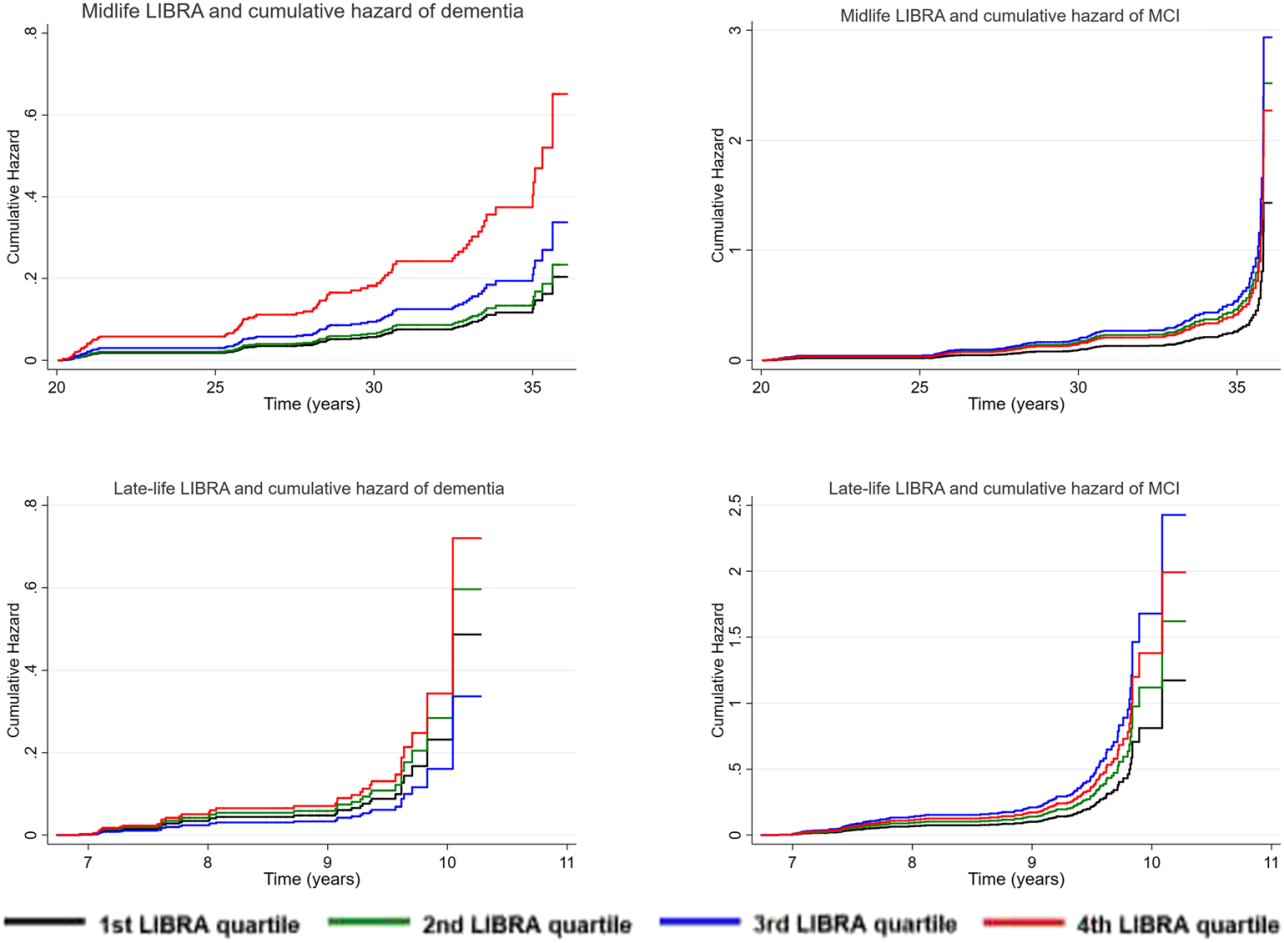
LIBRA score quartiles and cumulative hazard of dementia or mild cognitive impairment (MCI). Abbreviations: LIBRA, LIfestyle for BRAin health; MCI, mild cognitive impairment [Colour figure can be viewed at http://wileyonlinelibrary.com]

Performance of the LIBRA score in predicting dementia or MCI is shown in Table 2 (continuous LIBRA score) and Figure 2 (quartiles of LIBRA score). Higher midlife LIBRA scores were significantly related to higher dementia risk (hazard ratio [HR] = 1.27; 95% confidence interval [CI], 1.13‐1.43; C‐statistic = 0.67). In addition, higher midlife LIBRA scores were significantly associated with higher MCI risk (HR = 1.12; 95% CI, 1.03‐1.22; C‐statistic = 0.58), but not after adjustment for sex and education (HR = 1.05; 95% CI, 0.97‐1.16; C‐statistic = 0.65) (Table 2). When risk for dementia was categorized into quartiles (C‐statistic = 0.63), HRs for the second to fourth quartile (lowest quartile is the reference) were 1.15 (95% CI, 0.59‐2.23), 1.66 (95% CI, 0.89‐3.08), and 3.19 (95% CI, 1.78‐5.73), respectively. Quartile 4 predicted a higher dementia risk as compared with the lowest quartile (*P* < .001). For MCI (C‐statistic = 0.59), the second (HR = 1.76; 95% CI, 1.13‐2.74; *P* = .012) and third (HR = 2.05; 95% CI, 1.32‐3.19; *P* = .001) quartile predicted a higher MCI risk compared with the lowest quartile but not the fourth quartile (HR = 1.59; 95% CI, 0.95‐2.66; *P* = .078).

**Table 2:** Performance of the LIBRA score in predicting dementia and mild cognitive impairment (MCI)

	Dementia				MCI			
	n	HR	95% CI	Harrell C	n	HR	95% CI	Harrell C
**Midlife LIBRA**								
Model 1	1024	1.31*	1.17‐1.46	0.65	1024	1.12*	1.03‐1.22	0.58
Model 2	1019	1.27*	1.13‐1.43	0.67	1019	1.05	0.97‐1.16	0.65
Including diet, model 1	239	1.21	0.98‐1.50	0.68	239	1.01	0.86‐1.20	0.53
Including diet, model 2	231	1.14	0.89‐1.46	0.75	231	1	0.84‐1.20	0.69
**late‐life LIBRA**								
Model 1	604	1.01	0.84‐1.23	0.50	604	1.14*	1.02‐1.27	0.60
Model 2	595	1.02	0.84‐1.24	0.53	595	1.11*	1.00‐1.25	0.63

*Note*. Cox regression models with age as time scale and baseline age as origin. Model 1 is the unadjusted model. Model 2 is adjusted for sex and education.

Abbreviations: CI, confidence interval; HR, hazard ratio; LIBRA, LIfestyle for BRAin health; MCI, mild cognitive impairment.

* Values are statistically significant (*P* < .05).

In the small group of participants with available midlife diet data, the LIBRA score including diet was not significantly related to dementia or MCI risk, although C‐statistic values increased (Table 2). No significant interactions between midlife LIBRA score and APOE ε4 carrier status were found in any of the models (results not shown).

### 3.3 Late‐life LIBRA and incident dementia and MCI

Late‐life LIBRA scores were not significantly related to dementia. However, higher late‐life LIBRA scores were significantly associated with MCI risk (HR = 1.11; 95% CI, 1.00‐1.25; C‐statistic = 0.63). When risk for dementia was categorized into quartiles (C‐statistic = 0.58), none of them predicted a higher dementia risk as compared with the lowest quartile (second quartile: (HR = 1.23; 95% CI, 0.51‐2.93); third quartile: (HR = 0.69; 95% CI, 0.24‐2.00); fourth quartile: (HR = 1.48; 95% CI, 0.61‐3.61). For MCI (C‐statistic = 0.59), only the third quartile (HR = 2.07; 95% CI, 1.23‐3.47; *P* = .006) predicted a higher MCI risk compared with the lowest quartile but not the second (HR = 1.38; 95% CI, 0.79‐2.42; *P* = .260) and fourth quartile (HR = 1.69; 95% CI, 0.96‐3.00; *P* = .069).

Significant interactions between late‐life LIBRA scores and APOE ε4 carrier status were found in relation to dementia risk (*P* = .011). The HRs (95% CI) for dementia were 1.26 (0.96‐1.64) among non‐carriers, and 0.73 (0.53‐1.01) among carriers. No significant interactions between late‐life LIBRA scores and APOE ε4 carrier status were found in relation to MCI risk.

In late life, higher modified LIBRA scores (excluding midlife risk factors obesity and hypertension) were not significantly associated with dementia (HR = 1.22; 95% CI, 0.97‐1.53) or MCI (data not shown). Again, significant interactions between modified late‐life LIBRA scores and APOE ε4 carrier status were only found in relation to dementia risk (data not shown).

## 4 DISCUSSION

In a general Finnish population, higher midlife LIBRA scores were related to higher risk of developing dementia or MCI up to 30 years later. Higher late‐life LIBRA scores were related to higher risk of MCI up to 10 years later and to higher dementia risk, although the latter was restricted to APOE ε4 non‐carriers. These findings emphasize the potential role of modifiable risk factors in the development of MCI and dementia, and the potential usefulness of LIBRA as a tool for facilitating preventive strategies.

The LIBRA score was designed to reflect an individual's prevention potential for dementia and to identify individuals who might benefit from preventive strategies, and thus focuses exclusively on modifiable risk and protective factors, yielding a weighted sum score. Available risk scores for MCI or dementia have usually combined modifiable risk and protective factors with, eg, age, sex, formal education, cognitive performance, and/or other biomarkers.^29^ Age and education are strong risk factors for dementia and usually have the highest weights in risk scores they are part of.^30^ It is perhaps not surprising that the dementia predictive performance of the LIBRA score was lower than C‐statistic or area under the curve (AUC) values reported for such risk scores (eg, 0.75‐0.77 for the validated midlife CAIDE Dementia Risk Score versus 0.65 for midlife LIBRA score in the present study).^31^ The CAIDE risk score is based on a data‐driven approach within the CAIDE cohort study and is developed to maximize prediction of persons at‐risk of dementia.^32^ The C‐statistic improved after adding diet to the LIBRA score, although the small number of participants with midlife diet data, and lack of late‐life diet data limited these analyses. The lack of data on cognitive activity in the present study may also have affected the LIBRA score predictive performance, given the growing evidence that high engagement in cognitive activities is associated with lower risk for cognitive impairment or dementia.^6^

Higher midlife LIBRA scores were significantly related to higher dementia risk even after taking age, education, and sex into account, thus emphasizing the importance of modifiable lifestyle and vascular/metabolic factors for dementia risk, and the importance of early preventive strategies. Given the low awareness in the general public and health care professionals about links between such modifiable factors and dementia risk,^33^, ^34^, ^35^, ^36^ a great deal is to be gained and the LIBRA score could be useful for educational and motivational purposes to facilitate lifestyle changes for healthy cognitive aging and dementia prevention. LIBRA can give people insight and feedback on their personal risk profile and identify areas of already healthy behaviors (to facilitate maintenance), areas of unhealthy behaviors (to facilitate change), and chronic vascular/metabolic conditions (to facilitate appropriate management).^21^ This personal LIBRA profile is supported by an overview of risk factors with the highest penetrance (the accompanying weights/percentages are visualized in a clear manner) in relationship with their dementia risk. In this way, an individual gets an impression of the relative importance of each factor within their personal dementia risk profile. It can be used as a tool in clinical setting (eg, general practice) by helping people to make multiple intermediate steps (eg, reducing the score from +5 to +1) towards a more brain‐healthy lifestyle.^37^ Further, LIBRA can be used not only as a selection tool to identify high risk groups for future early intervention studies focused on dementia risk reduction but also as a surrogate/intermediate endpoint and surveillance tool to monitor intervention success along the road.

In the present study, performance of the midlife LIBRA score was better than the performance of the late‐life LIBRA score, suggesting that the significance of the selected modifiable risk and protective factors for dementia and MCI may be different in midlife compared with older ages. This is in line with previous studies showing that dementia risk scores based on midlife risk profiles tend to perform less well in older populations.^11^, ^29^, ^38^, ^39^ For example, important midlife vascular risk factors such as hypertension, obesity, or hypercholesterolemia tend to be less predictive for dementia at older ages,^3^, ^22^, ^23^, ^24^ and some late‐life risk scores have even included low blood pressure and/or low BMI as predictors.^29^ Blood pressure, BMI, and cholesterol tend to decline from midlife to late‐life in people who develop dementia later on, although the exact mechanisms are not fully clear.^5^ Such changes during the long pre‐clinical phase of dementia‐related diseases are also a major challenge for longer‐term dementia risk monitoring. The LIBRA score is also not very suitable for identifying individuals who are already close to dementia onset^38^ and who may need referral to specialized clinics for detailed cognitive and other assessments, as well as early initiation of pharmacological treatment.

The midlife LIBRA score performed better in predicting dementia risk than MCI risk. This is perhaps not surprising considering the notable heterogeneity of MCI as defined by older criteria in use at the time of the CAIDE visits. The performance of the late‐life LIBRA scores in predicting MCI risk (C‐statistic = 0.60) was similar to the basic prediction model developed in the Mayo Clinic Study of Aging, including education and self‐reported memory complaints together with several lifestyle and vascular factors.^40^

While the associations of midlife LIBRA scores with dementia or MCI risk were not influenced by the APOE ε4 allele in the present study, a significant interaction with APOE ε4 carrier status was found for late‐life LIBRA index in relation to dementia risk. Higher late‐life LIBRA scores were associated with elevated dementia risk particularly among non‐carriers. While some midlife lifestyle/vascular risk factors have been reported to have a more pronounced detrimental impact on dementia risk among APOE ε4 carriers compared with non‐carriers,^5^ it is possible that the impact of such risk factors among carriers may become less pronounced at older ages. APOE ε4 carriers who survive and do not develop dementia until after the age of 75 to 80 years represent a highly selected group where other genetic and/or non‐genetic risk and protective factors may also be important.

The major strengths of the present study include the population‐based design, the long follow‐up period starting already in midlife, and detailed assessments at several time points during the second half of the life‐course. For these reasons, CAIDE is a highly suitable cohort study for external validation of LIBRA. The risk prediction in this study applies only to individuals who actually survive to older ages, when they are more likely to develop dementia. As an inevitable part of all studies with a long‐follow up period, mortality, and non‐participation are often linked to poorer health, ie, people who are more likely to develop dementia, or die before dementia onset. This might have led to selection of a healthier sample and therefore may result in an underestimation of the “true” association. While data for most LIBRA factors were available in the CAIDE study, reliance on register diagnoses for some chronic conditions (ie, only conditions severe enough to require hospitalization) may have affected the predictive performance of the LIBRA score. Also, complete data on pharmacological treatment for the included risk factors/conditions were not available. In addition, interactions between risk factors were not taken into account in the design of the LIBRA score. Knowledge on possible interactions between risk factors is still incomplete in the available literature, and therefore more research on this matter is needed.

Findings from the present study emphasize the role of modifiable risk and protective factors in the development of MCI and dementia. The LIBRA score may be useful for educational and motivational purposes in public health initiatives by emphasizing areas amenable to preventive lifestyle measures and for identifying at‐risk individuals who may benefit from lifestyle interventions.

## Supporting information

Figure S1. Flow‐chart of the Cardiovascular Risk Factors, Aging and Dementia (CAIDE) study
Table S1. Definition of LIBRA factors in the CAIDE study

## Data Availability

According to the ethics rules and legislation in Finland, the individual level data cannot be made publicly available. Pseudonymised data can be made available with approval of the CAIDE Steering Committee (contact person Dr. Alina Solomon, alina.solomon@uef.fi).
