# Supplementary material for: Long‐term dementia risk prediction by the LIBRA score: A 30‐year follow‐up of the CAIDE study
Source: Int J Geriatr Psychiatry. 2019 Dec 6;35(2):195–203. doi: 10.1002/gps.5235 (PMC7003764; doi:10.1002/gps.5235)
Supplement: Supplementary file 1 — Figure S1. Flow‐chart of the Cardiovascular Risk Factors, Aging and Dementia (CAIDE) study Table S1. Definition of LIBRA factors in the CAIDE study [file GPS-35-195-s001.docx]

**Supplementary data**

**Figure 1.** Flow-chart of the Cardiovascular Risk Factors, Aging and Dementia (CAIDE) study

**1^st^ LATE-LIFE RE-EXAMINATION (1998)**

**n=1449**

Completed cognitive assessments n=1409

(Dementia n=61, MCI n=82)

Non-participants n=551 (poor health / refused / died)

Incomplete cognitive assessments n=40

**2^nd^ LATE-LIFE RE-EXAMINATION (2005-2008)**

**n=909**

Completed cognitive assessment n=852

(Dementia n=68, MCI n=171)

Not eligible n=574 (died, moved, unknown address)

Non-participants n=517 (poor health / refused / died)

Incomplete cognitive assessments n=57

**MIDLIFE (1972, 1977, 1982 or 1987)**

**n=2000**

Abbreviations: MCI, mild cognitive impairment; SBP, systolic blood pressure

**Table 1.** Definition of LIBRA factors in the CAIDE study

| **LIBRA factor*** | **Weight** | **CAIDE midlife examination** | **CAIDE 1998 late-life examination** |
| --- | --- | --- | --- |
| **Hypertension** | +1.6 | SBP≥140 mmHg or DBP≥90 mmHg; BP measured from subject’s right arm after seated down for five minutes | |
| **Obesity** | +1.6 | BMI ≥30 kg/m^2^ | |
| **High cholesterol** | +1.4 | - Serum total cholesterol ≥6.5 mmol/L (due to generally high levels in Eastern Finland and the CAIDE population)  - Cholesterol measurement methods differed in 1972 and 1977 (frozen serum, Lieberman-Burchard method) compared to later visits (fresh serum, enzymatic cholesterol oxidase/p-aminophenazone, CHOD-PAP, method). The systematic difference (2.4%) between methods was corrected accordingly. | |
| **Diabetes** | +1.3 | - Self-reported history of diabetes diagnosed by a physician, OR  - Diagnosis of diabetes recorded in the Finnish Hospital Discharge Register (ICD 8 code 250, ICD 9 code 250, ICD 10 codes E10-14); date of first diagnosis had to be before the date of the midlife examination (midlife LIBRA score), or the date of the 1998 re-examination (1998 LIBRA score) | |
| **Coronary heart disease** | +1.0 | - Self-reported history of myocardial infarction or angina diagnosed by a physician, OR  - Diagnosis of ischemic heart disease or atrial fibrillation recorded in the Finnish Hospital Discharge Register (ICD 8 codes 410-414 and 427.92, ICD 9 codes 410-414 and 4273A, ICD 10 codes I20-25 and I48); date of first diagnosis had to be before the date of the midlife examination (midlife LIBRA score), or the date of the 1998 re-examination (1998 LIBRA score) | |
| **Chronic kidney disease** | +1.1 | - Diagnoses recorded in the Finnish Hospital Discharge Register (ICD 8 code 582 for chronic nephritis; ICD 9 codes 585 for chronic renal failure and 791.0 for proteinuria; ICD 10 codes N18 for chronic renal failure and R80 for proteinuria). Date of first diagnosis had to be before the date of the midlife examination (midlife LIBRA score), or the date of the 1998 re-examination (1998 LIBRA score)  - Self-reports or creatinine measures not available | |
| **Physical inactivity** | +1.1 | Physical activity was assessed at all CAIDE examinations with the question: ‘How often do you participate in leisure-time physical activity that lasts at least 20-30 minutes and causes breathlessness and sweating?’ Response options were 1=daily; 2=2-3 times a week; 3=once a week; 4=2-3 times a month; 5=a few times a year; and 6=not at all. Options 3-6 were categorized as ‘physically inactive’, options 1-2 as ‘physically active’. | |
| **Low/moderate alcohol intake** | -1.0 | The following question was asked only for the 1972 and 1977 midlife cohorts (n=1464 of the 2000 CAIDE target population): ‘Do you use alcohol? How often on average do you consume wine or other alcoholic beverages?’. Response options were: 1=I do not use alcohol; 2=a couple of times/year or less; 3=3-4 times/year; 4=about once every 2 months; 5=about once/month; 6=a couple of times/month; 7=once/week; 8=a couple of times/week; 9=daily. | The following question was asked in 1998: ‘How often on average do you consume wine or other alcoholic beverages?’. Response options were: 1=at least once a month; 2=less than once a month; 3=not at all, I stopped consuming alcohol; 4=I do not use alcohol. |
|  |  | To keep the same definition at both midlife and 1998 examinations, low/moderate alcohol intake was defined as options 2-4 (midlife) and option 2 (1998). | |
| **Smoking** | +1.5 | Based on the question ‘Have you ever smoked during your life?’ with response options yes / no. | |
| **Depression** | +2.1 | A measure of hopelessness was used in the present study based on the following two items: 1) “I feel it is impossible to reach the goals that I would like to strive for”, and 2) “The future seems hopeless to me, and I can’t believe that things are changing for the better”. Participants were asked to rate their agreement/disagreement with each statement using a five-point Likert scale: 0-absolutely agree, 1-somewhat agree, 2-cannot say, 3-somewhat disagree, or 4-absolutely disagree. These items have previously been used in several population-based studies.^1^ The two items were combined, and points were given if participants had replied ‘somewhat agree’ or ‘absolutely agree’ to both items. | |
| **Healthy diet** | -1.7 | Based on the previously published CAIDE Healthy Diet Index.^2^ Range from 0 (minimal adherence to healthy diet) to 17 (maximal adherence). High adherence to healthy diet defined as>8 points. Only available in a limited number of participants. | Data not available |
| **High cognitive activity** | -3.2 | Data not available | Data not available |

*All factors are dichotomized as yes/no

BMI, body mass index; BP, blood pressure; CAIDE, Cardiovascular Risk Factors, Aging and Dementia; DBP, diastolic blood pressure; ICD, International Classification of Diseases; LIBRA, LIfestyle for BRAin health; SBP, systolic blood pressure

**References**

1. Everson SA, Goldberg DE, Kaplan GA, et al. Hopelessness and risk of mortality and incidence of myocardial infarction and cancer. Psychosom Med 1996;58:113-121.
2. Eskelinen MH, Ngandu T, Tuomilehto J, Soininen H, Kivipelto M. Midlife healthy-diet index and late-life dementia and Alzheimer's disease. Dement Geriatr Cogn Dis Extra 2011;1:103-112.
